# Retraction: Antibiotic Prescribing Behavior of Physicians in Outpatient Departments in Hospitals in Northwest Ethiopia: Structural Equation Modeling Approach

**DOI:** 10.2196/105480

**Published:** 2026-08-18

**Authors:** JMIR Publications Editorial Office

**Affiliations:** 1 JMIR Publications Toronto

The JMIR Publications Editorial Office is retracting the article “Antibiotic Prescribing Behavior of Physicians in Outpatient Departments in Hospitals in Northwest Ethiopia: Structural Equation Modeling Approach” [1] due to concerns regarding the integrity of the peer review process.

Author Asrat Agalu Abejew responded, stating that there were no conflicts of interest with the reviewers. However, the Editorial Office has determined that there is sufficient evidence indicating that the peer review process was compromised to proceed with a retraction.

Author Asrat Agalu Abejew disagreed with the decision to retract the article. The other authors did not respond to communications.
